# Identification of quantitative trait loci of agronomic traits in bread wheat using a Pamyati Azieva × Paragon mapping population harvested in three regions of Kazakhstan

**DOI:** 10.7717/peerj.14324

**Published:** 2022-11-09

**Authors:** Akerke Amalova, Kanat Yermekbayev, Simon Griffiths, Saule Abugalieva, Adylkhan Babkenov, Elena Fedorenko, Aigul Abugalieva, Yerlan Turuspekov

**Affiliations:** 1 Institute of Plant Biology and Biotechnology, Almaty, Kazakhstan; 2 Faculty of Biology and Biotechnology, Al-Farabi Kazakh National University, Almaty, Kazakhstan; 3 The John Innes Centre, Norwich, United Kingdom; 4 A.I. Barayev Research and Production Centre of Grain Farming, Shortandy, Kazakhstan; 5 North Kazakhstan Agricultural Experimental Station, Petropavlovsk, Kazakhstan; 6 Kazakh Research Institute of Agriculture and Plant Industry, Almalybak, Kazakhstan

**Keywords:** *Triticum aestivum* L, Quantitative trait loci, Mapping population, Recombinant inbred lines

## Abstract

**Background:**

Although genome-wide association studies (GWAS) are an increasingly informative tool in the mining of new quantitative trait loci (QTLs), a classical biparental mapping approach is still a powerful, widely used method to search the unique genetic factors associated with important agronomic traits in bread wheat.

**Methods:**

In this study, a newly constructed mapping population of Pamyati Azieva (Russian Federation) × Paragon (UK), consisting of 94 recombinant inbred lines (RILs), was tested in three different regions of Kazakhstan with the purpose of QTL identification for key agronomic traits. The RILs were tested in 11 environments of two northern breeding stations (Petropavlovsk, North Kazakhstan region, and Shortandy, Aqmola region) and one southeastern station (Almalybak, Almaty region). The following eight agronomic traits were studied: heading days, seed maturation days, plant height, spike length, number of productive spikes, number of kernels per spike, thousand kernel weight, and yield per square meter. The 94 RILs of the PAxP cross were genotyped using Illumina’s iSelect 20K single nucleotide polymorphism (SNP) array and resulted in the identification of 4595 polymorphic SNP markers.

**Results:**

The application of the QTL Cartographer statistical package allowed the identification of 53 stable QTLs for the studied traits. A survey of published studies related to common wheat QTL identification suggested that 28 of those 53 QTLs were presumably novel genetic factors. The SNP markers for the identified QTLs of the analyzed agronomic traits of common wheat can be efficiently applied in ongoing breeding activities in the wheat breeding community using a marker-assisted selection approach.

## Introduction

Wheat is one of the three most important food crops in the world. It is the staple food for about 40% of the world’s population, as it is one of the most abundant sources of calories and protein in the diet, providing nearly 20% of the total dietary protein worldwide (Braun et al., 2010). World wide wheat production in 2020–2021 amounted to 776.5 million tons. The FAO’s prediction for wheat production in 2021–2022 is expected to be 769.6 million tons, 6.9 million tons less than the previous year’s outturn (Food and Agriculture Organization of the United Nations, 2022). The main global exporters of wheat are Argentina, Australia, Canada, Europe, Kazakhstan, the Russian Federation, Ukraine, and the United States (Food and Agriculture Organization of the United Nations, 2022). Kazakhstan is one of the top ten bread wheat producers and exporters in the global market (USDA, 2019; Food and Agriculture Organization of the United Nations, 2022). According to the FAO, wheat production in Kazakhstan in 2021–2022 is expected to reach 12.0 million tons (Food and Agriculture Organization of the United Nations, 2022). In order to continue providing the world’s population with enough wheat in 2050, its yield should be increased by 60% (Shiferaw et al., 2013). Therefore, the constant improvement of productivity and quality is essential for wheat breeding (Li et al., 2019).

Productivity, stress resistance, and quality are complex traits controlled by many genes and genetic factors located in different regions of the chromosomes. Traits that are very important but are difficult to select by phenotype can be mapped or identified using appropriate phenotypic, genotypic, and statistical analyses. The underlying genetic architecture of a quantitative trait can be described by identifying a set of quantitative trait loci (QTLs) in the genome for a population and assigning effect values to these loci using an appropriate statistical model framework (Nettelblad, Mahjani & Holmgren, 2013). There are different methodologies for detecting and analyzing the presence of these loci (Collard et al., 2005; Xu et al., 2017).

Association mapping (AM) or a genome-wide association study (GWAS), which involves the use of diverse germplasm, has much higher resolving power in comparison to biparental linkage-based mapping (QTL mapping) and is currently considered the method of choice to unravel and understand the genetics of yield and yield-related traits (Sukumaran et al., 2015; Zanke et al., 2015; Turuspekov et al., 2017; Wang et al., 2017; Anuarbek et al., 2020; Mir, Kumar & Shafi, 2021; Gahlaut et al., 2021; Muhammad et al., 2021). Despite this, the classic biparental mapping approach is still a powerful method for finding unique genetic factors associated with important agronomic traits in common wheat (Wen et al., 2017). There are several reviews describing the use of statistical methods for mapping quantitative trait loci (QTLs, the genes responsible for variation in quantitative traits) in experimental crosses (Zou & Zeng, 2008; Van Eeuwijk et al., 2010). The method allows the tracking of QTL segregation by detecting markers associated with the traits of interest and assessing the effects, number of QTLs, and their locations on the respective chromosomes. Genetic linkage maps play an important role in genomic studies, including QTL mapping, MAS, and map-based cloning (Zhang & Gai, 2009).

Genetic maps of common wheat have progressed with the development of different types of DNA markers, to high-throughput marker systems recently used in wheat (Wang et al., 2014; Perez-Lara et al., 2016; Rasheed et al., 2017). There are a number of studies on QTL mapping using different genetic linkage maps of hexaploid wheat starting from the International Triticeae Mapping Initiative (ITMI) map, elaborated from the crosses of synthetic wheat W7984 (Altar 84 durum wheat x *Ae. tauschii*) and common wheat cultivar Opata 85, and a number of other maps (Paillard et al., 2003; Quarrie et al., 2005; Ma et al., 2015; Perez-Lara et al., 2016; Liu et al., 2020).

The construction of biparental mapping includes the choice of parents that differ in traits of interest, selection of molecular markers that distinguish between the two parents, development of a mapping population, genotyping and phenotyping of the mapping population, and identification of QTLs using a suitable statistical method (Xu et al., 2017). Prior to relating the trait to loci, genetic components of the trait of interest should be quantified through creating new recombination events. This can be achieved through the development of segregating populations. In this regard, RIL populations have been widely used in QTL mapping studies. For instance, RILs have successfully been used for the identification and validation of QTLs underpinning the traits of agronomic importance in staple cereals such as wheat (Börner et al., 2002; Griffiths et al., 2012; Ma et al., 2014; Ma et al., 2015; El-Feki et al., 2018; Onyemaobi et al., 2018; Tshikunde et al., 2019; Tura et al., 2020; Hu et al., 2020), Additionally, QTL studies of traits such as lodging (Yu & Chen, 2013), grain hardness (Sourdille et al., 1996), grain protein content (Prasad et al., 2003) and thousand grain weight (Varshney, Korzun & Börner, 2004), early heading (Xu et al., 2005), yield and its components (Kumar et al., 2007; Li et al., 2014; Shi et al., 2017; El-Feki et al., 2018; Onyemaobi et al., 2018; Tshikunde et al., 2019; Kumar et al., 2019; Tura et al., 2020; Hu et al., 2020; Ren et al., 2021), drought tolerance (Duggan, Domitruk & Fowler, 2000; Tahmasebi et al., 2017; Du et al., 2020) and disease resistance (Saini et al., 2022; Genievskaya et al., 2019; Genievskaya et al., 2020) in wheat were mainly based on the use of RILs. Importantly, the recently published wheat reference genome (Appels et al., 2018) allows us to retrieve the list of genes within these QTL intervals, identify their physical genomic coordinates and conduct functional annotation analysis. Thus, QTL mapping in crops has become a common practice due to the advances made in the area of molecular markers as well as that statistical genomics. QTL mapping approaches will need to acknowledge the central roles of QTL by environment interactions (QEI) and QTL by trait interactions in the expression of complex traits such as yield.

Earlier, QTL mapping for yield and its components, grain quality, and drought and disease resistance in Kazakhstan has been successfully performed using the DH mapping populations (MPs) Chinese Spring × SQ1 (Quarrie et al., 2005; Abugalieva, 2007; Abugalieva et al., 2010; Abugalieva et al., 2014) and Avalon × Cadenza (Amalova et al., 2021a). Still, the further development of local MPs is an important part of the local wheat breeding programs. Here we studied a RIL MP of Pamyati Azieva × Paragon (PAxP) developed within the framework of the international project “ADAPTAWHEAT” (Adaptawheat, 2012). Previously, the PAxP population was genotyped with KASP technology, where a low-density genetic map was constructed (Yermekbayev et al., 2020). Moreover, this population was successfully tested for resistance to leaf and stem rusts, both at the seedling and adult plant-growth stages, where 24 quantitative trait loci (QTLs) were identified for resistance to rust diseases (Genievskaya et al., 2019; Genievskaya et al., 2020). In addition, the PAxP mapping population showed a wide range of variation in yield-associated traits in southeast Kazakhstan (Amalova et al., 2019). The objective of this study was to identify QTLs for key agronomic traits using the population PAxP tested in three different environments of Kazakhstan during five years of trials, 2015–2020.

## Materials & Methods

### Plant materials

For this study, we used a bread wheat biparental MP from the cross of the spring wheat cultivars Pamyati Azieva (Russian Federation) and Paragon (United Kingdom), hereafter referred to as PAxP. The PAxP MP consisted of 94 recombinant inbred lines (RILs) and their development was described earlier (Amalova et al., 2019). The cultivar Pamyati Azieva was derived from the cross Saratovskaya 29 × Lyutestsens 99/80 − 1.7 and provided a combination of drought resistance, resistance to powdery mildew, and contained a high number of kernels per spike, which ensures high productivity per spike. The cultivar was recommended for the Western Siberian region of the Russian Federation and was approved for commercial cultivation in North Kazakhstan (Official website of the State Commission for Cultivars Testing of Crops of the Ministry of Agriculture of the Republic of Kazakhstan, 2022). Paragon was developed using the crosses CSW-1724-19-5-68//AXONA/TONIC and served both conventional and organic growers well and is recognized as a bread-making cultivar (UK recommended list, group 1). Moreover, Paragon has broad-spectrum disease resistance and good straw characteristics. The cultivar is a high-quality, bread-making UK spring wheat, does not contain the GA-insensitive *Rht-B1b* or *Rht-D1b* alleles, and is photoperiod sensitive (Kowalski et al., 2016).

### Evaluation of the MP for variation in agronomic traits

The studied traits were divided into two groups: plant adaptation-related traits and yield components. The plant adaptation traits included heading date (HD, days), seed maturation days (SMD, days), and plant height (PH, cm). The yield components included spike length (SL, cm), number of productive spikes (NPS, pcs), number of kernels per spike (NKS, pcs), thousand kernel weight (TKW, g), and yield per square meter (YM^2^, g/m^2^).

The MP and parents were evaluated in the experimental plots of Kazakhstan at the Kazakh Research Institute of Agriculture and Plant Industry (KRIAPI, Almaty, Southeast Kazakhstan) in 2015–2020, the A.I. Barayev Research and Production Centre for Grain Farming (RPCGF, Shortandy, North Kazakhstan) in 2018–2020, and the North Kazakhstan Agricultural Experimental Station (NKAES, Petropavlovsk, North Kazakhstan) in 2018–2019 (Supplemental File). The MP lines and parents were planted in two replications at each location in completely randomized blocks of 1 m^2^ plot. The distance between rows was 15 cm, with a five cm distance between plants (Dospekhov, 1985). The climate conditions recorded during the trials are shown in Table 1.

**Table 1:** Location, environment, and weather data at three regions in Kazakhstan.

Site/Region	**Almaty (Southeast of Kazakhstan)**						**Petropavlovsk (North)**		**Shortandy (North)**		
Latitude/Longitude	43°21′ / 76°53′						69°31′/4°10′		51°40′/71°00′		
Altitude, m	740						141		363		
Soil type	Light chestnut (humus 2.0–2.5%)						Black soil (humus 4.5–5%)		Southern carbonate chernozem (humus 3.6%)		
Conditions	Rainfed						Rainfed		Rainfed		
Year	2015	2016	2017	2018	2019	2020	2018	2019	2018	2019	2020
Annual rainfall, mm	228	550	301	217	299	279	272	151	321	430	426
Mean temperature, °C	21.4	19.9	20.8	19.6	19.8	19.8	16.1	16.3	15.2	17.1	19.2
Max temperature, °C	27.3	24.0	27.0	25.2	27.0	24.2	20.8	21.0	19.8	23.8	20.7
Min temperature, °C	16.8	16.0	14.7	14.8	12.9	14.2	10.8	10.2	9.8	12.4	17.6

### Genotyping and genetic map construction of the mapping population, QTL analysis, and statistics

The RILs and two parental cultivars were genotyped using Illumina’s iSelect 20K single nucleotide polymorphism (SNP) array at TraitGenetics (TraitGenetics GmbH, Gatersleben, Germany). The genotypic data were filtered to remove markers with >10% missing data, with <0.1 minor allele frequency, and markers with distorted segregation. In the end, the genetic map consisted of 4,595 polymorphic high-quality SNP markers and initially constructed by TraitGenetics GmbH (http://www.traitgenetics.com (Gaterseleben, Germany)) using JoinMap v5.0 software package (Stam, 1993) within the ADAPTAWHEAT project (Adaptawheat, 2012) funded by 7th European Union program. All SNPs showed a good fit to 1:1 segregation in the RILs mapping population (*p* > 0.001 in the Chi-squared test) (Genievskaya et al., 2019). To plot a haplotype map of the PAxP MP, the R statistical platform (R Studio Team, 2020) was applied.

The QTL analysis was performed using data from every year and location and the QTL identification was conducted as previously described in Amalova et al. (2021a) and Amalova et al. (2021a). Specifically, the composite interval mapping (CIM) method of Windows-based QTL Cartographer v2.5 software (Wang, Basten & Zeng, 2012), with the logarithm of the odds ratio (LOD) threshold of 3.0 was applied. The genetic map was drawn by MapChart v2.32 software (Voorrips, 2002). For the search for protein-coding genes that overlap with identified significant SNPs on the peak, each marker’s sequence was inserted into the BLAST tool of Ensembl Plants (2022) and compared with the Wheat Chinese Spring IWGSC RefSeq v1.0 genome (Appels et al., 2018). Proteins were identified using the UniProt database (UniProt, 2022) *via* cross-reference from Ensembl Plants (2022). Pearson’s correlation and three-way analysis of variance (ANOVA) analyses were performed using the R statistical platform (R Studio Team, 2020). ANOVA analysis was performed based on data for two years (2018 and 2019) in three studied locations. The broad-sense heritability (H^2^) was calculated according to Covarrubias-Pazaran (2019). The Additive Main Effect and Multiplicative Interaction (AMMI) analysis and Finley–Wilkinson analysis were analyzed using GenStat software (VSN International, 2019).

## Results

### Genetic map

**Figure 1:**
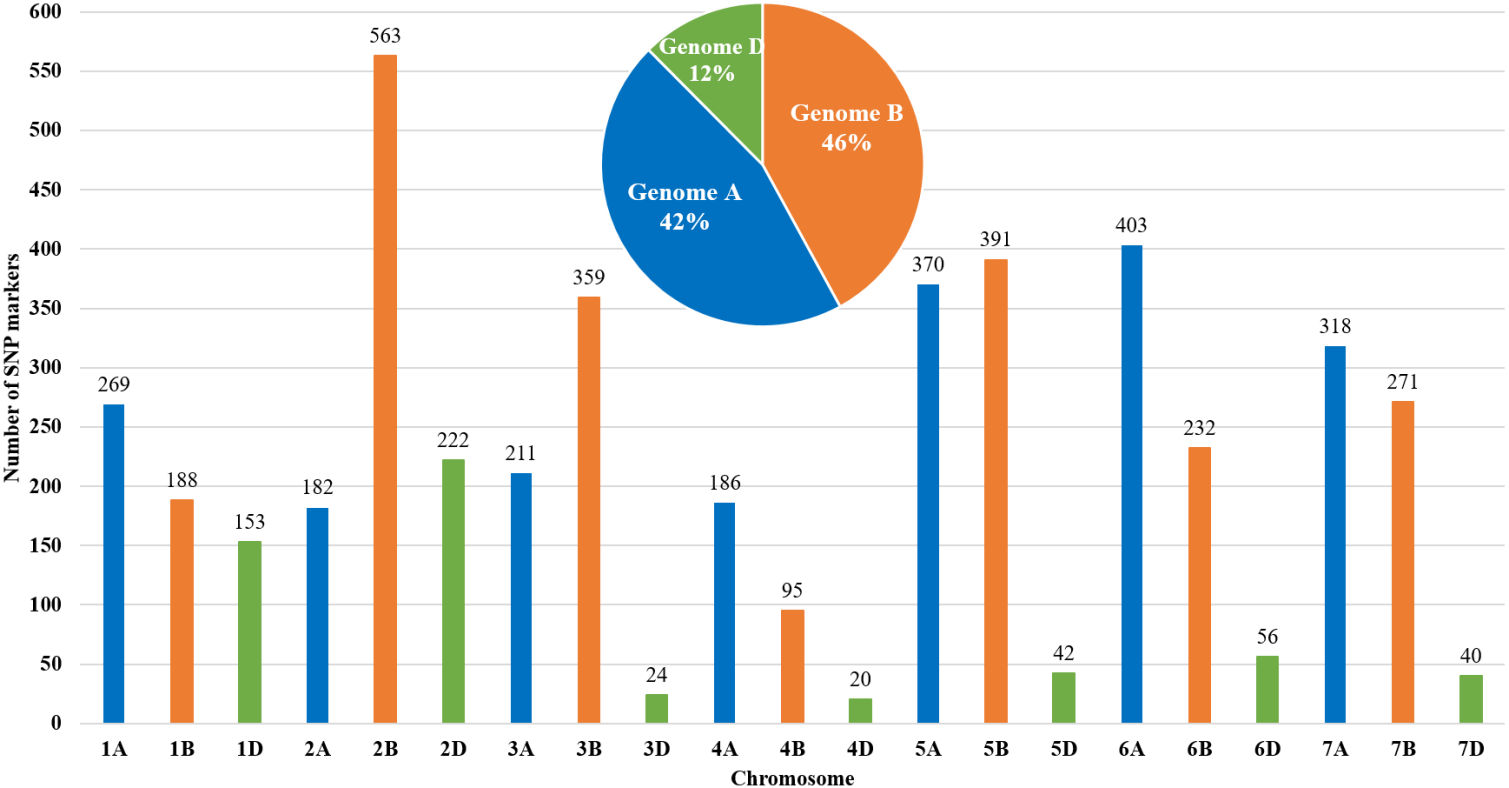
Distribution of 4,595 single nucleotide polymorphisms (SNPs) across 21 chromosomes in 94 RILs of the bread wheat Pamyati Azieva × Paragon mapping population.

The genetic map of bread wheat Pamyati Azieva × Paragon was constructed by using 94 RILs of the MP and 4,595 polymorphic SNP markers (Fig. 1). The total length of the genetic map was 2,723.90 cM with a mean intermarker distance of 1.60 cM. Chromosome 2B contained the largest number of markers at 563, having a length of 150.6 cM, and a mean distance of 3.74 cM between markers. Chromosome 4D had the smallest number, with only 20 markers, as the length of this chromosome was 16.8 cM with a mean distance of 1.19 cM. The gap reflected the degree of linkage between the markers, ranging from 1.77 cM (chr 2B) to 8.28 cM (chr 3D), with a mean value of 3.50 cM (Table S1). Among informative 4,595 SNPs, few markers showed a slight segregation distortion towards either AA or BB alleles, but the *p*-values, obtained from the chi-square test of Mendelian segregation, remained to be statistically significant (Table S2). The assessment of allelic proportions for RILs provided that 46.7% derived from Pamyati Azieva (red) and 47.5% from Paragon (blue) with the remaining 5.8% being heterozygotes (green) (Fig. S1).

Altogether, of the 4595 high-quality polymorphic markers, 1939 (42.2%) markers were localized to the A genome, 2099 (45.7%) markers were mapped to the B genome, and 557 (12.1%) were mapped to the D genome (Fig. 1).

### Comparative assessment of phenotypic variations of the MP in three studied research organizations

The field experiments were conducted at three locations: (1) KRIAPI (2015–2020), (2) RPCGF (2018–2020), and (3) NKAES (2018–2019). RILs were studied at three locations, and the phenotypic trait variation indicated a large level of variability and significance among regions. The average HD varied from 42.3 ± 0.16 days at NKAES to 60.5 ± 0.15 days at KRIAPI. The average PH ranged from 92.0 ± 0.78 cm at KRIAPI to 55.7 ± 0.45 cm at RPCGF (Table 2). The average yield components (SL, NPS, NKS, and YM^2^) showed a high value at KRIAPI, followed by NKAES. However, the average TKW was higher at RPCGF and NKAES in comparison to KRIAPI (Table 2).

**Table 2:** The phenotypic variability of the agronomic traits in the RILs of the Pamyati Azieva × Paragon bread wheat mapping population that was tested in three locations in Kazakhstan.

Traits	Site	Check cultivar	Parental cultivars		RILs		
			Pamyati Azieva	Paragon	min	max	mean+SE
HD	NKAES	40.0	40.5	44.5	39.0	46.2	42.3 ± 0.16
	RPCGF	55.0	42.0	41.0	37.7	51.3	42.5 ± 0.18
	KRIAPI	51.0	59.7	58.7	57.2	66.0	60.5 ± 0.15
SMD	NKAES	42.8	46.5	47.3	38.5	49.3	45.1 ± 0.19
	RPCGF	59.0	56.0	57.0	54.3	61.0	56.5 ± 0.11
	KRIAPI	40.7	36.7	34.7	14.7	38.0	35.4 ± 0.20
PH	NKAES	89.7	81.3	66.3	61.5	91.8	76.4 ± 0.62
	RPCGF	60.9	55.2	54.3	44.3	65.7	55.7 ± 0.45
	KRIAPI	103.3	95.9	91.4	61.7	106.9	92.0 ± 0.78
SL	NKAES	9.3	10.3	8.8	7.8	12.4	9.6 ± 0.08
	RPCGF	8.4	8.6	8.9	7.6	10.8	9.0 ± 0.07
	KRIAPI	10.2	11.0	11.0	8.7	13.8	11.0 ± 0.10
NPS	NKAES	2.8	2.9	2.6	1.6	3.8	2.4 ± 0.04
	RPCGF	2.8	2.0	1.9	1.1	2.9	1.7 ± 0.04
	KRIAPI	5.1	3.8	4.0	2.5	5.3	3.7 ± 0.05
NKS	NKAES	35.1	32.0	35.3	27.6	43.7	34.8 ± 0.37
	RPCGF	30.9	37.5	34.8	27.7	42.5	35.8 ± 0.35
	KRIAPI	45.5	46.1	57.7	40.5	60.5	49.4 ± 0.50
TKW	NKAES	33.8	32.1	30.5	28.4	42.5	35.8 ± 0.27
	RPCGF	33.5	34.2	32.9	25.6	44.9	34.6 ± 0.34
	KRIAPI	40.9	31.3	28.2	22.4	36.2	30.3 ± 0.26
YM2	NKAES	278.7	317.3	221.4	133.4	310.6	226.5 ± 4.15
	RPCGF	300.6	294.9	251.3	73.7	464.3	271.0 ± 7.91
	KRIAPI	619.4	483.6	578.5	207.4	666.9	519.8 ± 11.80

**Notes.**

The experimental station NKAES (Petropavlovsk), RPCGF (Shortandy), KRIAPI (Almaty region). Astana was used as a check cultivar in the North regions (NKAES and RPCGF), and Kazakhstanskaya 4 in the southeast region (KRIAPI).

The three-way ANOVA for genotypes was significantly different between two-factor regions and years for seven traits (Table S3). The analysis showed a significant difference between two-factor (genotype, region) for HD (2.02), NPS (1.49), and NKS (1.36). Also was calculated heritability (H^2^) for all traits in studied years in locations (Table S3).

### Correlation of phenological traits of the Pamyati Azieva × Paragon bread wheat MP in the three studied regions

**Figure 2:**
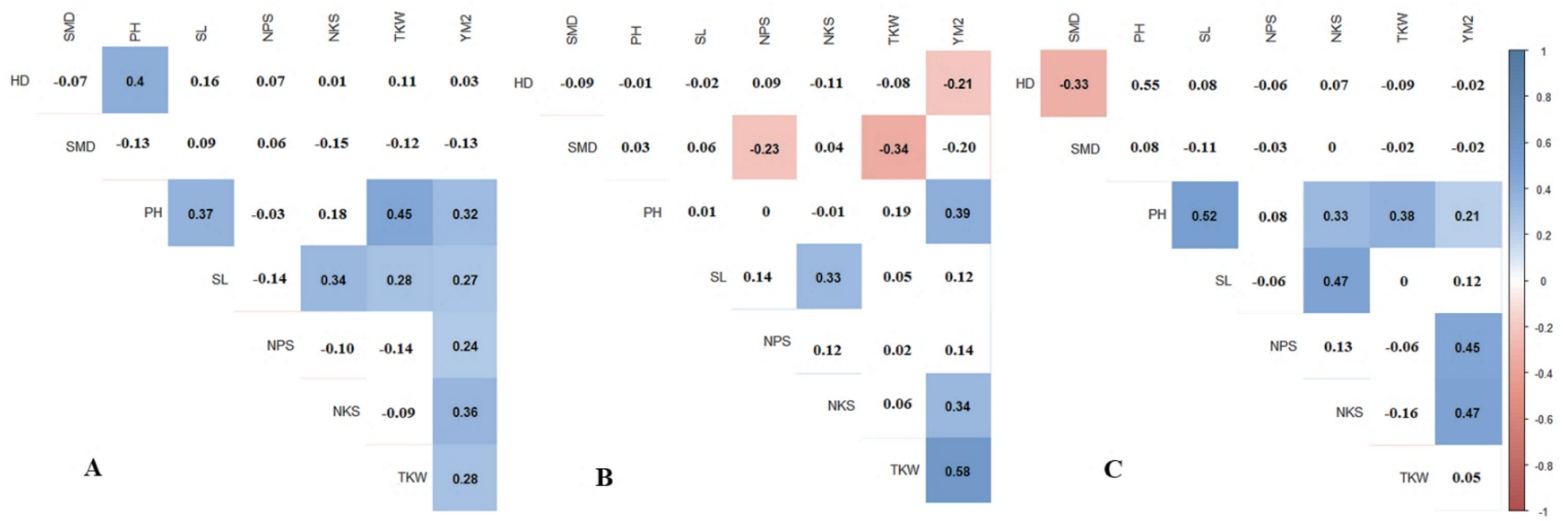
Pearson’s correlation index based on multiple years’ data in three regions: (A) NKAES (Petropavlovsk); (B) RPCGF (Shortandy); and (C) KRIAPI (Almaty). Correlations with *P* < 0.05 are highlighted in color. The color indicates either positive (blue) or negative (red) correlations.

Pearson’s correlation results suggest that in RPCGF, HD was negatively correlated with YM^2^, while in NKAES and KRIAPI, this association was not observed (Fig. 2B). At all three testing sites, average values of NKAES, RPCGF, and KRIAPI, PH was positively correlated with YM^2^. NKS was significantly associated with the yield at all three sites, with the highest average values recorded in KRIAPI (0.47), followed by NKAES (0.34) and RPCGF (0.33) (Fig. 2). In contrast, the average value of TKW over two years (2019–2020) was correlated with YM^2^ at the two northern stations but not in the southeastern region (Table S4). The correlation of NKS and TKW was in congruence with the results in Table 2, as the highest average NKS was recorded at KRIAPI, and TKW at the two northern stations was significantly higher than that at KRIAPI.

### TKW and YM^2^ assessment of individual RILs in the three locations

TKW and YM^2^ are important agronomic traits in the evaluation of wheat performance in the field. Overall, 39, 7, and 19 RILs demonstrated higher YM^2^ in comparison to the appropriate check cultivars in NKAES, RPCGF, and KRIAPI, respectively (Fig. S2). Moreover, three lines (PAxP-01, PAxP-05, and PAxP-28) demonstrated high productivity in Northern Kazakhstan. In addition, five highly productive lines (PAxP-07, PAxP-14, PAxP-16, PAxP-20, and PAxP-21) were revealed at two sites (Shortandy and Almaty). Two lines (PAxP-01 and PAxP-05) showed high productivity for YM^2^ in all three regions (Fig. S2). The TKW assessment showed a wide distribution range. The analysis of the average value of TKW revealed 21 and 32 RILs that exceeded the TKW of the check cultivars in the Astana in the Shortandy and Petropavlovsk regions, respectively (Fig. S3). In addition, eight lines (PAxP-04, PAxP-66, PAxP-68, PAxP-82, PAxP-84, and PAxP-92) showed higher values compared to the local check cultivar Astana in Northern Kazakhstan.

The TKW and YM^2^ results showed that lines PAxP-01, PAxP-04, PAxP-05, PAxP-68, PAxP-82, and PAxP-84 were highly productive in comparison to the check cultivars in the three regions. Particularly, the Finley–Wilkinson analysis suggested that PAxP-01 and PAxP-05 showed highly productive YM^2^ across all three tested sites, and PAxP-05 showed a very stable, high TKW (Fig. S4).

### Assessment of AMMI the MP in the three studied regions

The Additive Main Effects and Multiplicative Interaction (AMMI) analysis of average YM^2^ well-separated all three studied environments. PC1 (Principal Component 1) was particularly informative (68.04%) and was the largest contributor to the separation between two regions (north *vs* southeast), while PC2 (31.96%) effectively separated NKAES from the RPCGF region (Fig. S5).

The assessment of the AMMI plot for YM^2^ showed the highest average yield performance was at KRIAPI. The RILs located in the center of the plot showed high stability in the three studied regions. Several RILs, including PAxP-21, PAxP-07, PAxP-11, and PAxP-35, showed higher yield performances at KRIAPI and RPCGF (Fig. S5). Four RILs (PAxP-72, PAxP-65, PAxP-92, and PAxP-93) demonstrated a high average yield at KRIAPI and NKAES but not at RPCGF (Fig. S5).

### QTL mapping of agronomic traits in north and southeast regions

**Figure 3:**
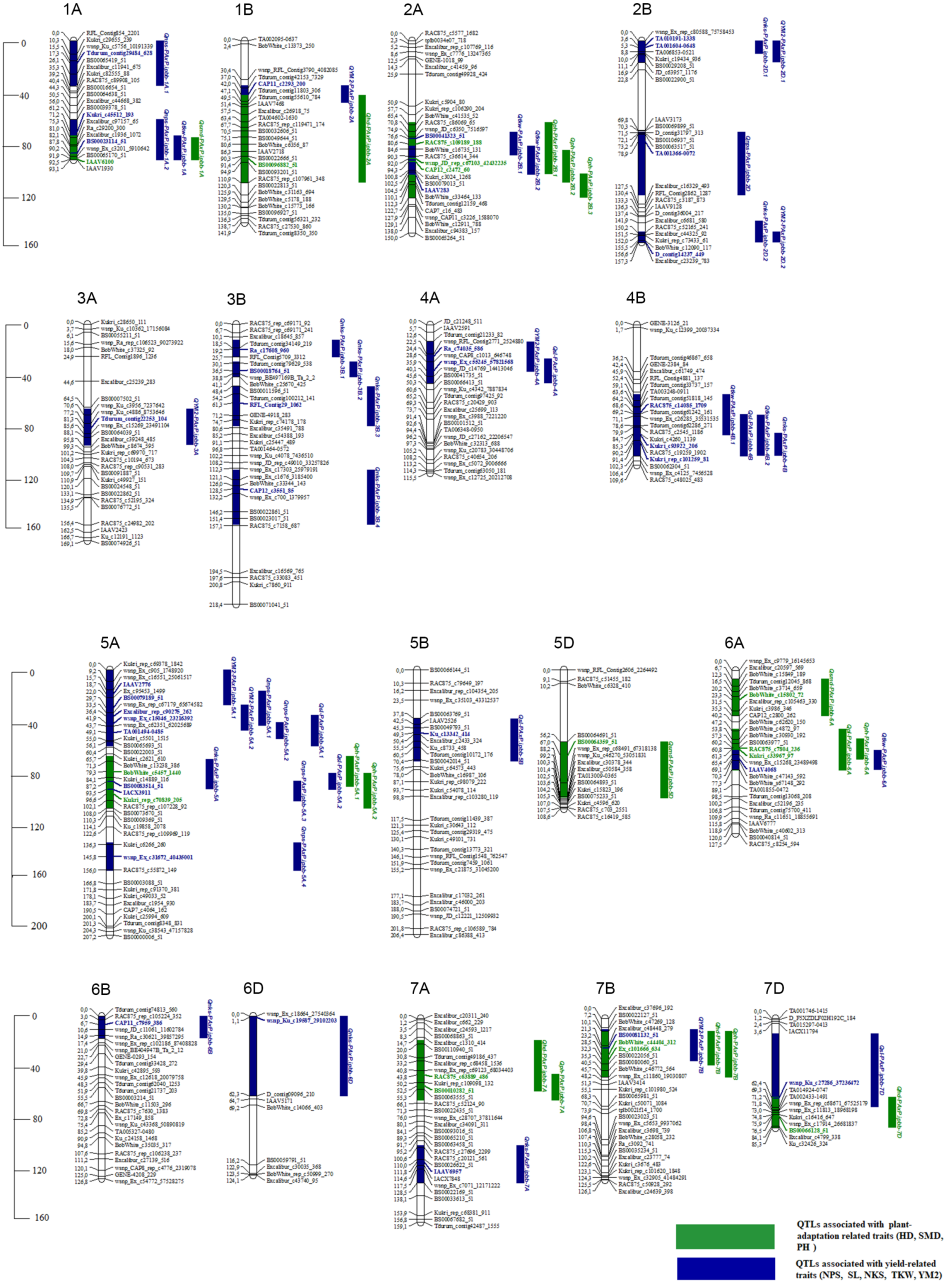
The genetic map of QTLs associated with plant adaptation and yield components and identified using the Pamyati Azieva × Paragon mapping population. The marker’s names are shown on the right, and marker loci positions are shown on the left of the linkage maps in centimorgans (cM). Significant markers in identified QTLs were given in blue for traits NPS, SL, NKS, TKW, and YM2 and green for HD, SMD, and PH.

The results of the QTL analysis of the 94 RILs of mapping population PAxP identified 53 stable QTLs for eight traits out of the detected 296 QTLs. The QTL LOD ranged from the threshold value of 3 (*Qhd-PAxP.ipbb-7D, Qnps-PAxP.ipbb-1A.1,* and *Qnps-PAxP.ipbb-5A.1*) to 10.9 (*Qsl-PAxP.ipbb-4A*),and located on 17 chromosomes. Among the eight traits, the number of identified QTLs ranged from 3 QTLs for SMD to 11 QTLs for NKS (Table S5). The total number of stable QTLs identified for the group of plant adaptation-related traits was fifteen (Table S5 and Fig. 3), and the number of QTLs for the group of traits for yield components was thirty-eight (Table S5 and Fig. 3).

### QTLs for the yield-related traits (NPS, SL, NKS, TKW, and YM^2^)

The stable QTLs for the group of yield components varied from 6 QTLs (SL and TKW) to 11 QTLs (NKS). The R^2^ varied between 10% (*Qnps-PAxP.ipbb-2D* and *QYM*^2^*-PAxP.ipbb-4A*) to 40% (*Qnks-PAxP.ipbb-6D*). The highest LOD was 10.9, which detected *Qsl-PAxP.ipbb-4A* (Table S5 and Fig. 3).

Seven QTLs were associated with NPS. All QTLs were located on chromosomes 1A (two QTLs), 2D, and 5A (four QTLs). Six QTLs were associated with SL. The QTLs for SL were detected on chromosomes 4A, 4B, 5A (two QTLs), 5B, and 7D. Two QTLs (*Qsl-PAxP.ipbb-4B* and *Qsl-PAxP.ipbb-5A.1*) were detected in Northern Kazakhstan (RPCGF and NKAES). *Qsl-PAxP.ipbb-4A* was detected in the two regions (Almaty and Shortandy) that had LOD values ranging from 3.4 to 10.9 (Table S5 and Fig. 3).

The highest number of QTLs was detected for NKS. Eleven QTLs for NKS were identified and mapped on chromosomes 2D (two QTLs), 3B (four QTLs), 4B, 5A, 6B, 6D, and 7A. The two QTLs located on chromosome 2D and two QTLs located on chromosome 3B were detected in southeast Kazakhstan from 2015 to 2019. The three QTLs (*Qnks-PAxP.ipbb-3B.4, Qnks-PAxP.ipbb-4B,* and *Qnks-PAxP.ipbb-6B*) located on the 3B, 4B, and 6B chromosomes were identified only at NKAES. Seven QTLs showed an additive effect of between 1.63 pcs and 4.11 pcs with the origin of Pamyati Azieva. *Qnks-PAxP.ipbb-6D* was detected in the two regions (Almaty and Shortandy), showed the highest R^2^ (40%), and was the donor of increasing alleles from Paragon (−5.22 pcs) (Table S5 and Fig. 3).

**Figure 4:**
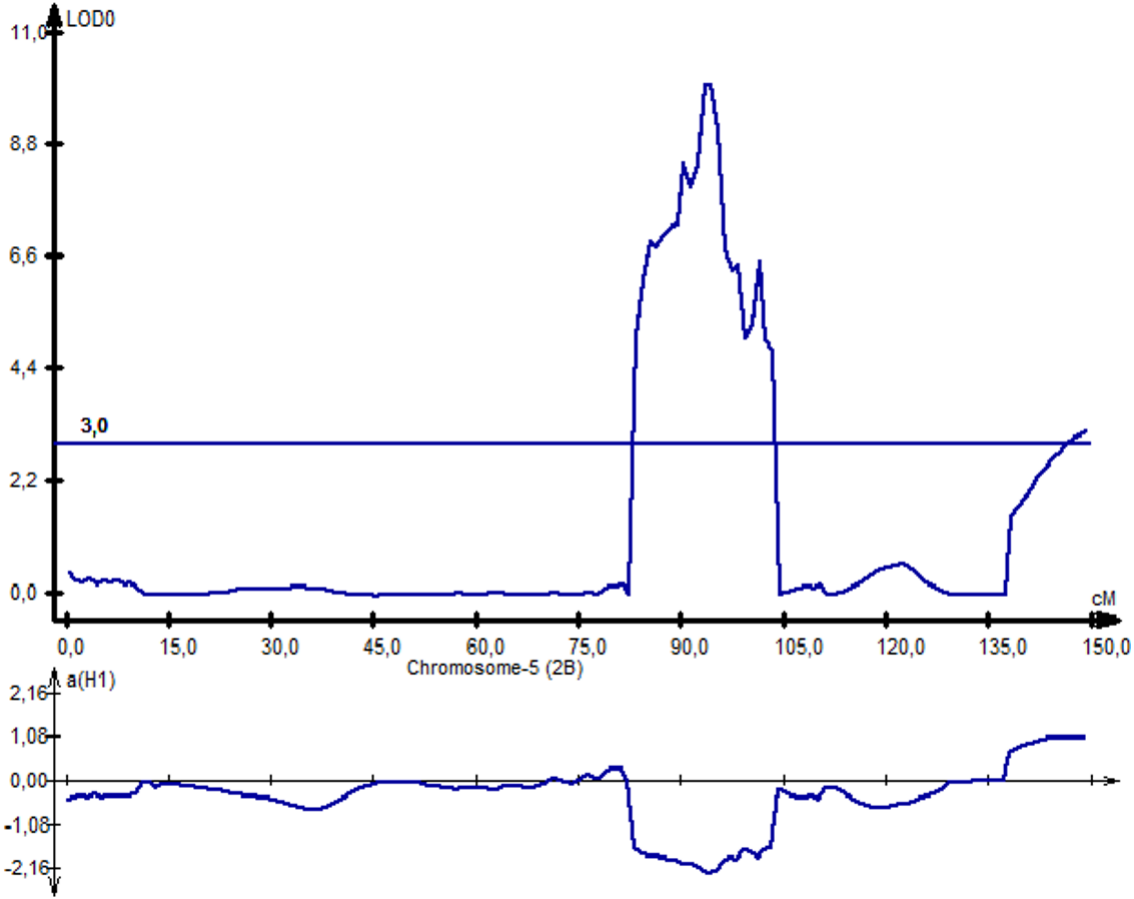
The quantitative trait locus *Qtkw-PAxP*. ipbb-2B.2 for the trait “thousand kernel weight” on chromosome 2B.

Following NKS, the next two traits with the largest identified numbers of QTL were YM^2^ (eight QTLs) and TKW (six QTLs). The assessment of the remaining QTLs for YM^2^ suggested that three loci (*QYM* ^2^*-PAxP.ipbb-2D.1, QYM*^2^*-PAxP.ipbb-4A,* and *QYM*^2^*-PAxP.ipbb-5A.2)* were common for two northern stations (RPCGF and NKAES). Likewise, *Qtkw-PAxP.ipbb-2B.2* for TKW (Fig. 4) was detected in two Northern Kazakhstan stations (RPCGF and NKAES) and showed the highest LOD value (9.9) and R^2^ (34%). Among all of the QTLs, *Qtkw-PAxP.ipbb-2B.1* showed the highest additive effects from Pamyati Azieva (3.85 g) (Table S5 and Fig. 4).

### QTLs for the plant adaptation-related traits (HD, SMD, and PH)

The QTLs identified in multiple environments for the group of plant adaptation-related traits varied from four QTLs (HD), three QTLs (SMD), and eight QTLs (PH). The R^2^ for each individual QTL varied between 12% (*Qph-PAxP.ipbb-7B*) and 26% (*Qhd-PAxP.ipbb-7A*). The highest LOD was 8.2, which was observed at *Qph-PAxP.ipbb-7A*. Among the QTLs, eight showed positive additive effects, with Pamyati Azieva increasing the effects of the QTLs, while seven had negative additive effects, with Paragon increasing the effects of the QTLs (Table S5 and Fig. 3).

Four QTLs were detected for HD, one each on chromosomes 2A, 7A, 7B, and 7D. The QTL *Qhd-PAxP.ipbb-7A* was also detected in the three regions and had LOD values from 3.1 to 8.1, with the additive effect from Paragon of −0.9 days. The second, *Qhd-PAxP.ipbb-7B*, was detected in the two regions and had LOD values ranging from 3.7 to 9.6, with an additive effect equaling 0.72 days from Pamyati Azieva as the donor of alleles. For the SMD, three QTLs were detected on chromosomes 1A, 5D, and 6A. Among the QTLs for the SMD, two showed additive effects from Pamyati Azieva and one additive effect from Paragon, increasing the effects of the QTLs in Northern Kazakhstan (Table S5 and Fig. 3).

**Figure 5:**
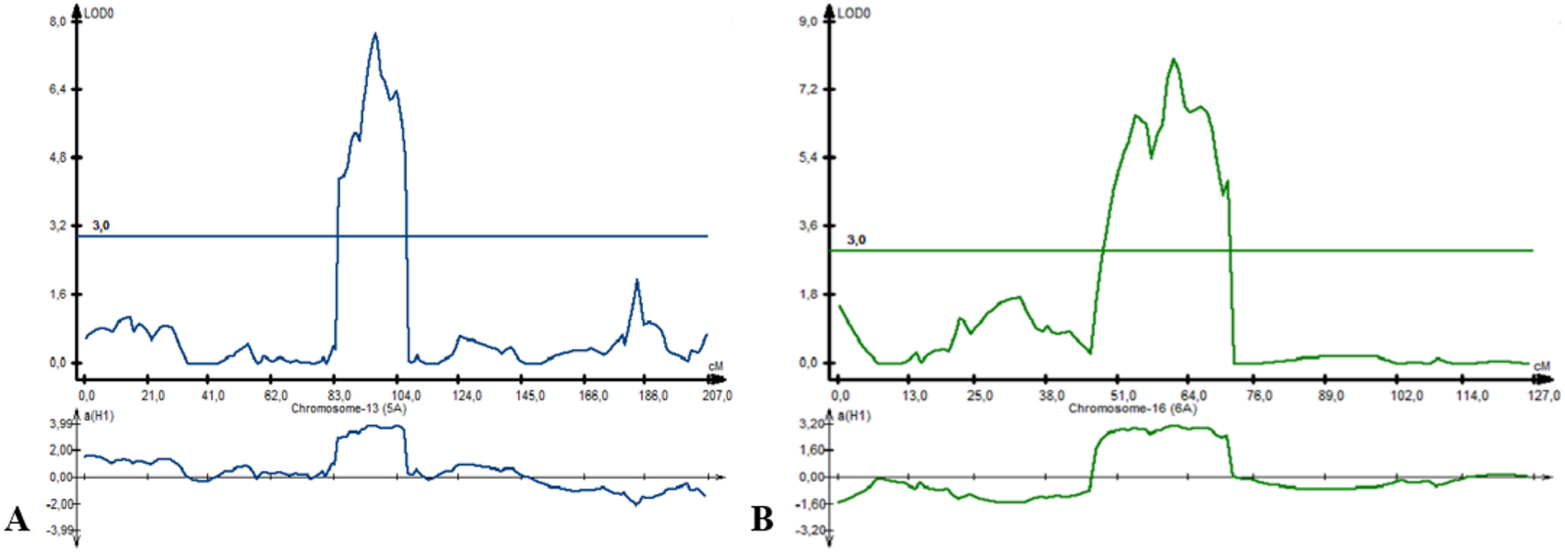
The position of identified quantitative trait loci (QTLs) for plant height (PH), which were revealed on chromosomes 5A (A) and 6A (B).

For PH, eight QTLs were detected on chromosome 2B (three QTLs), 5A (two QTLs), 6A, 7A, and 7B. Three QTLs (*Qph-PAxP.ipbb-2B.3, Qph-PAxP.ipbb-5A.1,* and *Qph-PAxP.ipbb-5A.2)* were identified only in Southeast Kazakhstan (KRIAPI). The two QTLs with the highest LOD values were *Qph-PAxP.ipbb-5A.2* and *Qph-PAxP.ipbb-6A,* which originated from Pamyati Azieva (Fig. 5). *Qph-PAxP.ipbb-7A* is the QTL with the highest LOD value (8.2).

The highest additive PH effect was observed for *Qph-PAxP.ipbb-2B.2* (height-reducing allele from Paragon), which decreased height up to 4.56 cm (Fig. 3 and Table S5). One of the QTLs for PH (*Qph-PAxP.ipbb-7B*) coincided with the position of *QYM*^2^*-PAxP.ipbb-7B*, which is the QTL for YM^2^ (Table S5 and Fig. 3). Notably, in all identified QTLs for PH, the decreasing alleles were from Paragon, and the increasing alleles were from Pamyati Azieva (Fig. 3 and Table S5).

**Figure 6:**
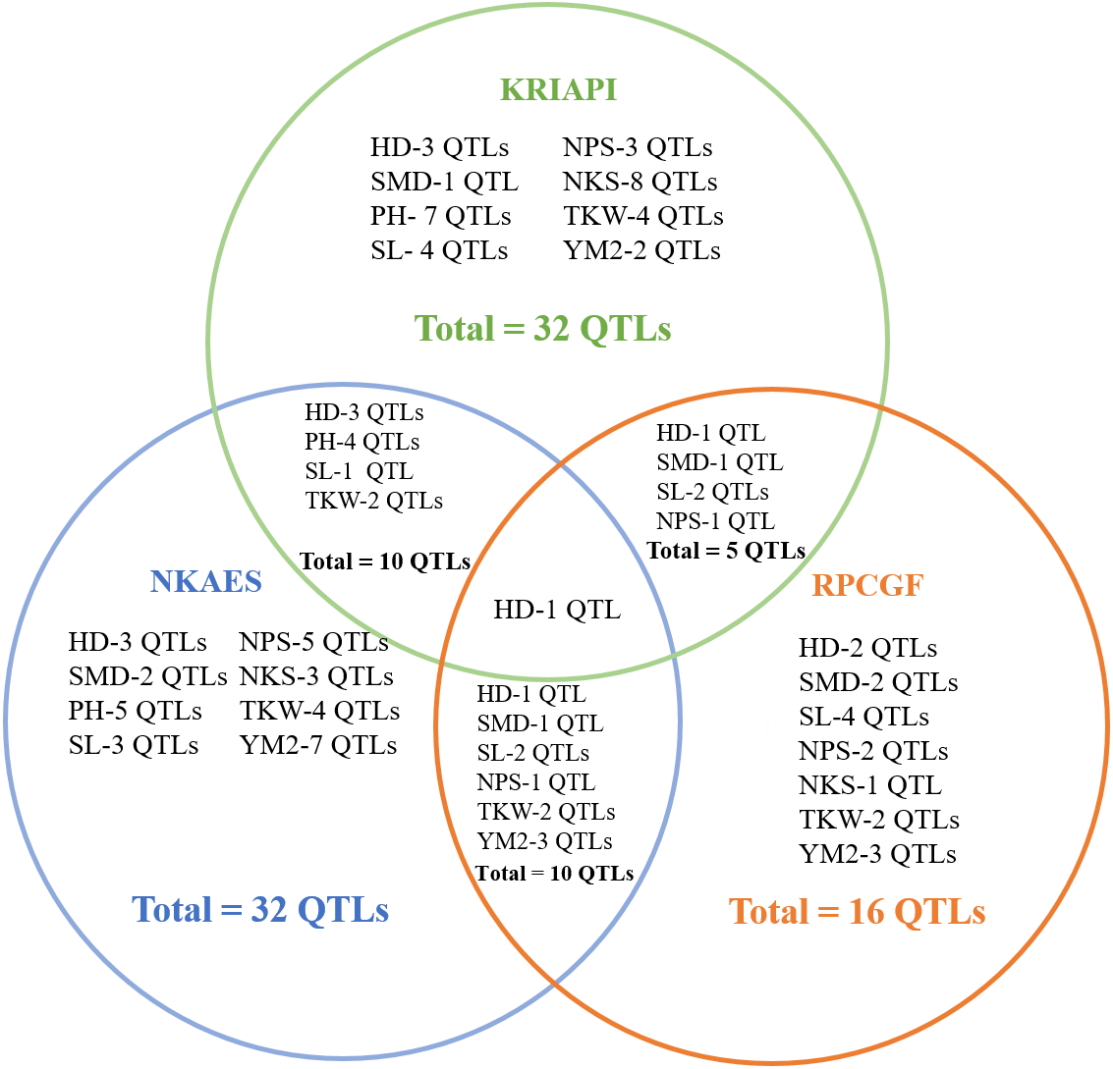
The number of stable quantitative trait loci (QTLs) for eight studied traits identified in three regions (NKAES, Petropavlovsk, in blue; RPCGF, Shortandy, in brown; KRIAPI, Almaty, in blue circles).

The total identified QTLs found in the three regions varied from 32 QTLs in Southeast Kazakhstan (KRIAPI), and 32 QTLs in (NKAES) and 16 QTLs (RPCGF) in Northern Kazakhstan (Fig. 6). Only one common QTL (*Qhd-PAxP.ipbb-7A*) among the studied traits was identified in the three regions. Ten common QTLs identified in Shortandy (RPCGF) and Petropavlovsk (NKAES) were common between these regions, and five common QTLs were identified in the Almaty (KRIAPI**)** and Shortandy (RPCGF) regions (Fig. 6). Three QTLs (*QYM* ^2^*-PAxP.ipbb-2D.1, QYM*^2^*-PAxP.ipbb-4A,* and *QYM*^2^*-PAxP.ipbb-5A.2*) were detected only in Northern Kazakhstan (RPCGF and NKAES).

## Discussion

### Identification of QTLs for agronomic traits using the Pamyati Azieva × Paragon mapping population

The success in the identification of new QTLs for yield components depends on many factors, such as well-developed MP, high-resolution genotyping of the MP, genetic and phenotypic variability of the population defined by selected parental lines, genotype-environment interaction patterns, etc. (Xu et al., 2017; Slafer, Savin & Sadras, 2014). In this study, the MP developed by using two genetically distinct parents bred in the UK and Russian Federation was genotyped based on 4,595 SNPs in all three subgenomes. The SNP genotyping of this MP provided higher resolution than in our latest QTL mapping effort using Avalon x Cadenza MP, which relied on 3,647 polymorphic SNPs (Amalova et al., 2021a). Moreover, whereas the Avalon x Cadenza doubled haploid MP was constructed using two British cultivars, the PAxP MP was developed using varietal representatives of two genetically distant pools that were tested in three wheat-growing regions of the country. Therefore, in this study, it was expected that a larger number of novel QTLs would be identified in comparison to the QTL mapping of eight agronomic traits using the Avalon x Cadenza MP.

The QTL mapping assessment using field trials of the PAxP in three contrasting regions allowed the detection of 53 QTLs identified in two or more environments (Fig. 3) out of a total of 296 QTLs for eight agronomic traits, suggesting that the remaining 243 QTLs were region-specific associations. Only one stable QTL (HD) was common among all three tested regions, confirming the sharp environmental differences in the selected sites (Fig. 6). It was found that, although the NKAES and RPCGF experimental sites belong to the Northern region of the country, the shared number of QTLs in these two sites (10 QTLs) was equal to those that were shared between KRIAPI (southeast region) and NKAES (Fig. 6). Evidently, the growing conditions in NKAES and KRIAPI better facilitated the higher variability in the studied traits, as the number of identified QTLs at the RPCGF was half that in the other two sites. At the same time, there were certain conditions, such as KRIAPI 2019, where relatively limited precipitation and lower temperature at the booting stage possibly affected yield components. This limitation also influenced the correlation between TKW and YM^2^ (Table S4), which is in normal conditions supposed to be positive one.

Interestingly, the only one common QTL for all three tested sites (*Qhd-PAxP.ipbb-7A)* was identified for HD and was located on chromosome 7A, with a peak of 43.2 cM (700,955,008 bp) (Table S6). the literature survey indicated that *Qhd-PAxP.ipbb-7A* (30,147,904-727,580,363 bp) was mapped in the vicinity of *Vrn-A3* (TaFTA), which was located at 45 cM and linked with a *barc154* (65,515,540 bp) microsatellite marker (Bonnin et al., 2008). Moreover, the genetic location of *Qhd-PAxP.ipbb-7A* coincided with a QTL for HD, which was located on 43.5 –46.5 cM (78,328,789–82,350,351 bp) (Hu et al., 2020). Therefore, it is possible that *Qhd-PAxP.ipbb-7A* is associated with *Vrn-A3* and influenced the flowering time of the MP by Paragon, as it was a source for the efficient allele in the association (Table S5).

Notably, the genetic position of one of those QTLs for PH (*Qph-PAxP.ipbb-7B*) was located on chromosome 7B on marker interval 10.1 cm–45.9 cm (3,701,651–115,373,417 bp) and matched the position of QTL for YM^2^. None of the detected QTLs for PH was found at the RPCGF site, which is an indication that conditions in this region were rather stressful and suppressed the mean values not only for PH but also for NPS (Fig. 3). This indicates that the variability of traits was reduced under the stress condition of RPCGF. The number of identified QTLs at the RPCGF sites was also lower in comparison to KRIAPI and NKAES for yield components, including NPS, NKS, and TKW (Fig. 6). A comparison of mapped QTLs for traits analyzed in this study *versus* previous studies, including those that were detected using Avalon x Cadenza MP, indicated that 28 QTLs were presumably novel genetic factors for the eight analyzed traits as 25 QTLs matched known associations (Table S6). The fact that 25 QTLs found in this study were also reported elsewhere is confirming (1) the functional importance of these marker-trait associations (MTAs) on a wide geographical scale, and (2) provides additional support for the robustness of findings in this study. The overview of similar genetic locations of QTLs for the same targeted traits shows that three QTLs for HD (*Qhd-PAxP.ipbb-2A, Qhd-PAxP.ipbb-7A,* and *Qhd-PAxP.ipbb-7B)* were identified a similar interval on the same chromosome in RILs study associated with yield and heading date in China (Hu et al., 2020; Chen et al., 2020). Within the region, four associations matched the results from studies of the CS × SQ1 DH mapping population, where QTLs for PH (*Qph-PAxP.ipbb-5A.2)*, SL (*Qsl-PAxP.ipbb-5A.2),* and NPS (*Qnps-PAxP.ipbb-1A.* and *Qnps-PAxP.ipbb-5A.3)* were identified in southeastern Kazakhstan (Quarrie et al., 2005; Abugalieva, 2007). The seven associations matched the results from studies of the UK reference mapping population Avalon × Cadenza*,* where QTLs associations with PH, SL, NPS, NKS, and TKW were identified in the northern, central, and southern regions of Kazakhstan (Amalova et al., 2021a). *Qnps-PAxP.ipbb-1A.2* was identical to the genetic positions of QTLs identified with the analyses of six traits using GWAS based on the assessment of common wheat in three different regions of Kazakhstan (Turuspekov et al., 2017). Two QTLs (*Qnps-PAxP.ipbb-5A.3* and *Qtkw-PAxP.ipbb-4B.2)* were identical to the same genetic position of QTLs identified in the GWAS of yield components in the spring wheat collection harvested under two water regimes in Northern Kazakhstan (Amalova et al., 2021b) (Table S6).

We have aligned the significant SNPs of identified 53 stable QTLs to the Chinese Spring reference genome (Appels et al., 2018) using the Wheat Ensembl database (Ensembl Plants, 2022). The results showed that out of 53 significant SNPs, 44 and nine QTLs were located in genic and intergenic genomic positions respectively (Table S5). Among, the alignment of the significant SNPs in the 28 identified presumably novel QTLs with sequences in the database showed that 15 SNPs in genic positions (Table S5). The five presumably novel QTLs were aligned with the basic helix-loop-helix transcription factors (*Qsl-PAxP.ipbb-4B)*, F-box domain-containing protein (*Qsl-PAxP.ipbb-5B, Qsmd-PAxP.ipbb-5D),* ubiquitin core domain-containing protein (*Qnks-PAxP.ipbb-3B.2),* E2 ubiquitin-conjugating enzyme (*Qsmd-PAxP.ipbb-1A).* The novel QTL for SL *(Qsl-PAxP.ipbb-4B)* was aligned with gene *TraesCS4B02G364900*, which codes protein basic helix-loop-helix transcription factors. The protein is expressed in the endosperm of the seed and also in the spikes during the heading time (Guo & Wang, 2017) (Table S4). The other two novel QTLs (*Qsl-PAxP.ipbb-5B, Qsmd-PAxP.ipbb-5D)* where significant SNPs were aligned with F-box domain-containing protein. It is known that F-box proteins regulated plant development and control flowering time (Jain et al., 2007; Hong et al., 2012). Another identified important alignment of novel QTLs and specific genes is related to a ubiquitin-associated group of enzymes. E3 ubiquitin-protein ligase, along with E1 ubiquitin-activating and E2 ubiquitin-conjugating enzymes, is known to participate in the ubiquitylation of proteins (Liu et al., 2020). Ubiquitylation itself is essential for the regulation of various biological processes, including growth and development, response to biotic and abiotic stress, and regulation of chromatin structure (Ramadan et al., 2015; Xu et al., 2021). In this study, we identified that the most significant SNPs in two QTLs (*Qnks-PAxP.ipbb-3B.2* and *Qsmd-PAxP.ipbb-1A*) were aligned with ubiquitin core domain-containing protein and an E2 ubiquitin-conjugating enzyme, respectively (Table S5).

### Evaluation of PAxP recombinant inbred lines in multiple tested environments

An additional value of an MP tested in multiple environments is the possibility of identifying promising RILs with high yield potential. Our previous analysis of Avalon x Cadenza in several regions of Kazakhstan allowed the express extraction of valuable genetic lines (Amalova et al., 2021a) that were instantly introduced into the selection processes of several breeding organizations. Similarly, it was anticipated that RILs generated from Paragon and Pamyati Azieva, two genetically very distant cultivars, would provide plenty of promising candidates with a high yield potential in three different regions of the country. As expected, 39, seven, and 19 RILs were identified with higher YM^2^ than standard cultivars in NKAES, RPCGF, and KRIAPI, respectively (Fig. S2). The assessment of those extracted RILs using Finley–Wilkinson testing was also helpful in identifying PAxP-05 and PAxP-01 as potentially valuable sources for high TKW and YM^2^ in all three regions. PAxP-05 and PAxP-01 showed outstanding YM^2^ performance in KRIAPI, in the average TKW values in the two northern stations, and in SL, NPS, and NKS data in all three studied regions (Table S7). In addition, PAxP-05 carried a positive allele for 32 of the identified QTLs, including those with high effects for HD, NPS, TKW, and YM^2^ (Table S7). Generally, each of identified RILs with outstanding field performance could be used as a donor to improve wheat adaptation and productivity in Kazakhstan. Thus, comprehensive means of genetic and analytical studies for the identification of new phenotypically stable alleles or/and allelic combinations for the traits of interest can be successfully used in local wheat breeding projects.

## Conclusions

The 94 RILs of the PAxP MP that were developed from genetically distant cultivars and were genotyped using Illumina’s iSelect 20K SNP array resulted in the identification of 4595 polymorphic SNP markers. The RILs were tested in 11 environments in two northern and one southeastern region of Kazakhstan and showed a wide range in yield performance. In total, 39, seven, and 19 RILs were identified with a higher average YM^2^ than standard cultivars in NKAES, RPCGF, and KRIAPI, respectively. Two RILs, PAxP-05 and PAxP-01, showed high average TKW and YM^2^ values in all three regions. The environmental patterns differently influenced the yield performance. For instance, Pearson’s correlation results suggested that HD was negatively correlated with YM^2^ in the RPCGF site, but the correlation was not significant in the NKAES and KRIAPI sites. In all three regions (NKAES, RPCGF, and KRIAPI), the average PH was positively correlated with YM^2^. The average NKS was significantly associated with the yield in all three regions, while average the TKW was correlated with YM^2^ only in the two northern regions (NKAES and RPCGF). The phenotypic data of RILs studied in 11 environments of the three regions were used for the identification of important QTLs associated with the studied agronomic traits. The application of the QTL Cartographer statistical package allowed for the identification of 53 stable QTLs (Table S5 and Fig. 3) out of a total of 296 QTLs for eight agronomic traits. A survey of published studies related to common wheat QTL identification suggested that 28 QTLs for the eight analyzed traits were presumably novel genetic factors, while 25 QTLs matched known associations. The findings in this study can be very helpful for further validation of identified MTAs for their use in bread wheat breeding projects for the development of new competitive and highly productive cultivars.

##  Supplemental Information

10.7717/peerj.14324/supp-1 Supplemental Information 1 Raw field data from three region of Kazakhstan

10.7717/peerj.14324/supp-2 Supplemental Information 2 The segregation of 4595 polymorphic SNP markers in mapping population Pamyati Azieva × Paragon consisting of 94 recombinant inbred lines

10.7717/peerj.14324/supp-3 Supplemental Information 3 Segregation ratio of 4595 polymorphic SNP markers in mapping population Pamyati Azieva × Paragon

10.7717/peerj.14324/supp-4 Supplemental Information 4 Three-way analysis of variance (ANOVA) for phenotypic traits of the Pamyati Azieva × Paragon

10.7717/peerj.14324/supp-5 Supplemental Information 5 Pearson’s correlation index based on studies in three regions: (1) NKAES (Petropavlovsk); (2) RPCGF (Shortandy); and (3) KRIAPI (Almaty)

10.7717/peerj.14324/supp-6 Supplemental Information 6 List of 53 stable QTLs identified in the Pamyati Azieva × Paragon mapping population

10.7717/peerj.14324/supp-7 Supplemental Information 7 List of identified QTLs based on field trials of the Pamyati Azieva × Paragon mapping population and comparative analyses with the associations revealed in previously published reports

10.7717/peerj.14324/supp-8 Supplemental Information 8 Evaluation of two RILs of mapping population Pamyati Azieva × Paragon (PAxP) in multiple tested environments

10.7717/peerj.14324/supp-9 Supplemental Information 9 Haplotype map of mapping population Pamyati Azieva × Paragon consisting of 94 recombinant inbred lines

10.7717/peerj.14324/supp-10 Supplemental Information 10 Scatter plot of MP for traits YM^2^ in the three regions A –Northern Kazakhstan, B –Southeast Kazakhstan

10.7717/peerj.14324/supp-11 Supplemental Information 11 Distribution of 94 RILs Pamyati Azieva × Paragon MP for TKW in the three regions: (A) NKAES (Petropavlovsk); (B) RPCGF (Shortandy); and (C) KRIAPI (Almaty)

10.7717/peerj.14324/supp-12 Supplemental Information 12 Finley–Wilkinson analysis of TKW (A) and YM^2^ (B) at the three tested sites. TKW—thousand kernel weight; YM^2^ –yield per square meter

10.7717/peerj.14324/supp-13 Supplemental Information 13 AMMI output plot for average values of YM^2^ in three regions: NKAES (Petropavlovsk), RPCGF (Shortandy), and KRIAPI (Almaty region) Environments are shown in blue and Genotypes in green color.

## Additional Information and Declarations

### Data Availability

The following information was supplied regarding data availability: The raw measurements are available in the Supplemental Files.
