# Supplementary figures and images for: Identification of quantitative trait loci of agronomic traits in bread wheat using a Pamyati Azieva × Paragon mapping population harvested in three regions of Kazakhstan
Source: PeerJ. 2022 Nov 9;10:e14324. doi: 10.7717/peerj.14324 (PMC9653069; doi:10.7717/peerj.14324)

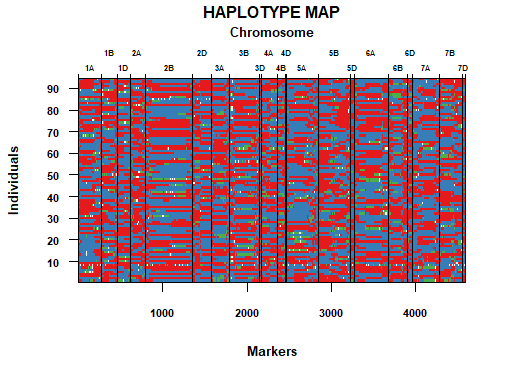

Supplement: Supplemental Information 9 [file peerj-10-14324-s009.png]

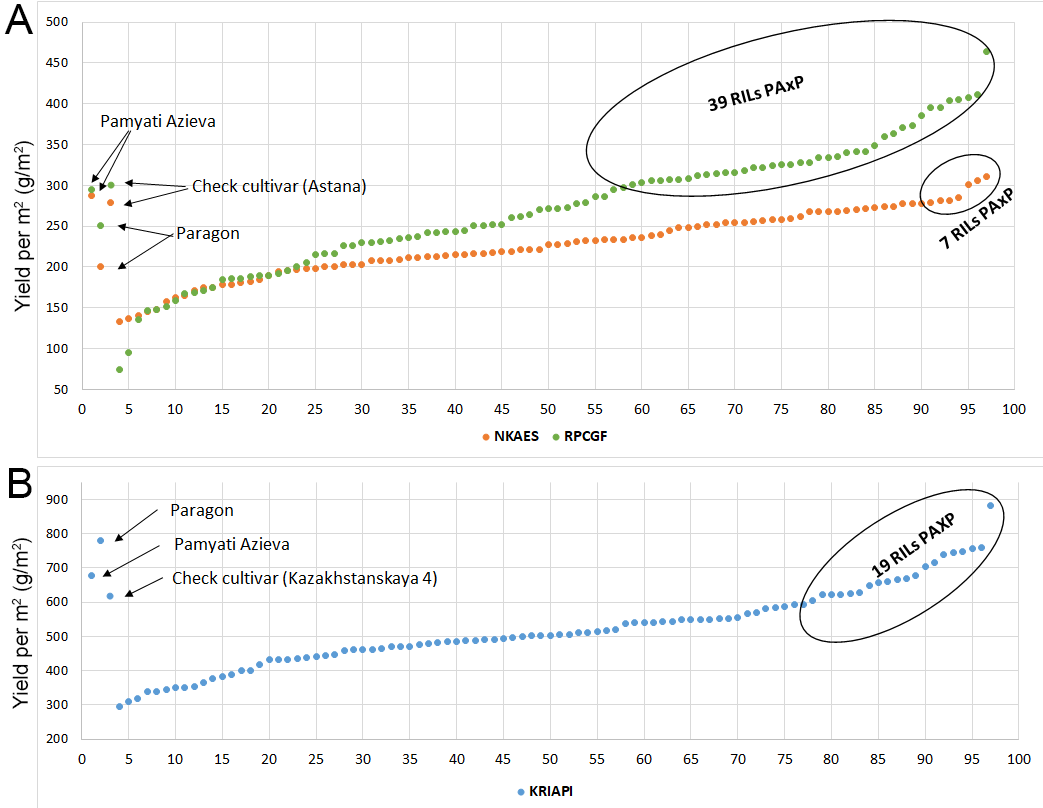

Supplement: Supplemental Information 10 [file peerj-10-14324-s010.png]

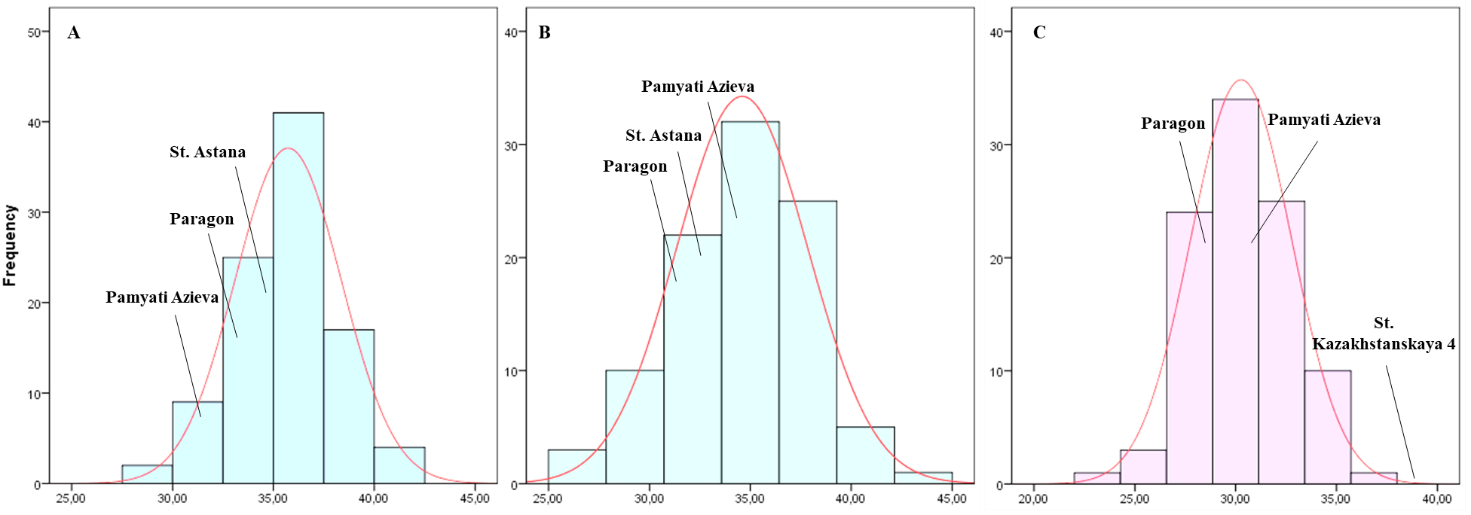

Supplement: Supplemental Information 11 [file peerj-10-14324-s011.png]

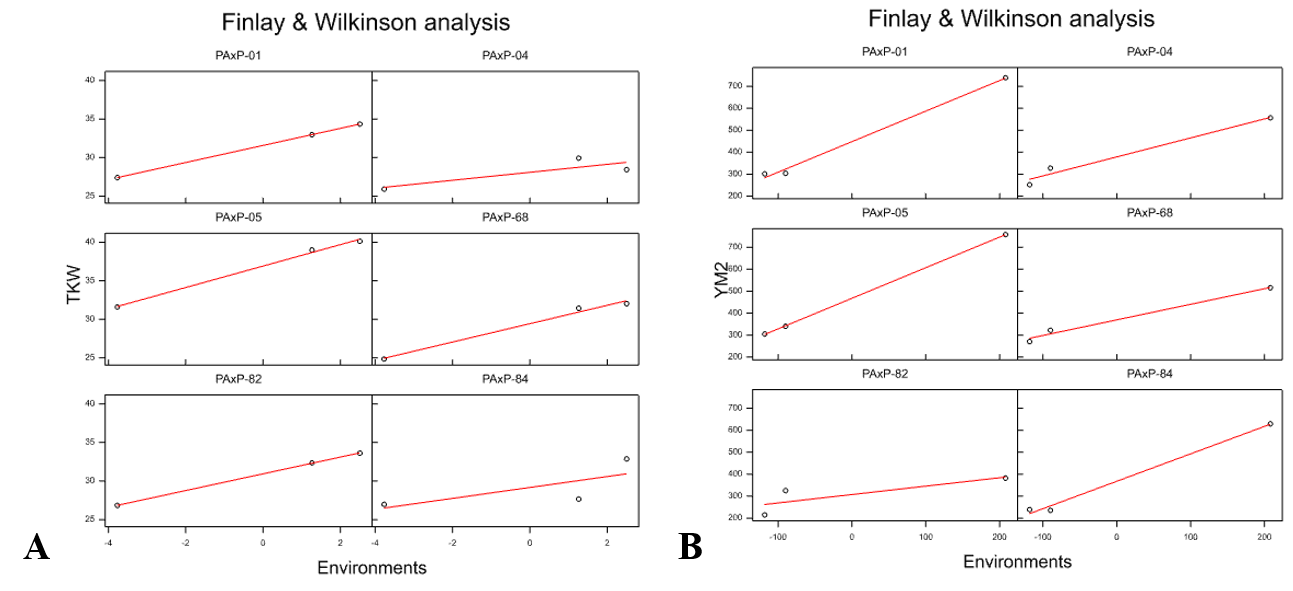

Supplement: Supplemental Information 12 [file peerj-10-14324-s012.png]

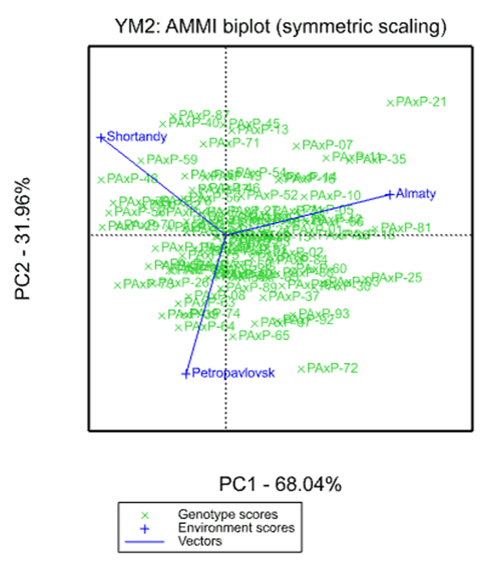

Supplement: Supplemental Information 13 — Environments are shown in blue and Genotypes in green color. [file peerj-10-14324-s013.png]
